# Drug discovery for heart failure targeting myosin-binding protein C

**DOI:** 10.1016/j.jbc.2023.105369

**Published:** 2023-10-20

**Authors:** Thomas A. Bunch, Piyali Guhathakurta, Andrew R. Thompson, Victoria C. Lepak, Anna L. Carter, Jennifer J. Thomas, David D. Thomas, Brett A. Colson

**Affiliations:** 1 Department of Cellular & Molecular Medicine, University of Arizona, Tucson, Arizona, USA; 2 Department of Biochemistry, Molecular Biology, and Biophysics, University of Minnesota, Minneapolis, Minnesota, USA; 3 Photonic Pharma LLC, Minneapolis, Minnesota, USA

**Keywords:** actin, cardiac muscle, cardiac myosin-binding protein C (cMyBP-C), contractile proteins, phosphorylation, protein kinase A (PKA), fluorescence lifetime (FLT), high-throughput screen (HTS), fluorescence resonance energy transfer (FRET), site-directed spectroscopy

## Abstract

Cardiac MyBP-C (cMyBP-C) interacts with actin and myosin to fine-tune cardiac muscle contractility. Phosphorylation of cMyBP-C, which reduces the binding of cMyBP-C to actin and myosin, is often decreased in patients with heart failure (HF) and is cardioprotective in model systems of HF. Therefore, cMyBP-C is a potential target for HF drugs that mimic its phosphorylation and/or perturb its interactions with actin or myosin. We labeled actin with fluorescein-5-maleimide (FMAL) and the C0-C2 fragment of cMyBP-C (cC0-C2) with tetramethylrhodamine (TMR). We performed two complementary high-throughput screens (HTS) on an FDA-approved drug library, to discover small molecules that specifically bind to cMyBP-C and affect its interactions with actin or myosin, using fluorescence lifetime (FLT) detection. We first excited FMAL and detected its FLT, to measure changes in fluorescence resonance energy transfer (FRET) from FMAL (donor) to TMR (acceptor), indicating binding. Using the same samples, we then excited TMR directly, using a longer wavelength laser, to detect the effects of compounds on the environmentally sensitive FLT of TMR, to identify compounds that bind directly to cC0-C2. Secondary assays, performed on selected modulators with the most promising effects in the primary HTS assays, characterized the specificity of these compounds for phosphorylated *versus* unphosphorylated cC0-C2 and for cC0-C2 *versus* C1-C2 of fast skeletal muscle (fC1-C2). A subset of identified compounds modulated ATPase activity in cardiac and/or skeletal myofibrils. These assays establish the feasibility of the discovery of small-molecule modulators of the cMyBP-C-actin/myosin interaction, with the ultimate goal of developing therapies for HF.

Cardiomyopathies are the most common and severe inherited disorders that are associated with significant adverse outcomes such as heart failure, arrhythmias, and sudden cardiac death (1). Hypertrophic cardiomyopathy (HCM) results from a mutation(s) in 11 or more different sarcomeric genes (2) and is characterized by hypertrophy of the left ventricle and the interventricular septum, typically reducing ventricular chamber volume and causing myocyte and myofibrillar disarray. In HCM, the heart typically becomes enlarged, hypercontractile, and unable to relax effectively, but the clinical manifestations of the disease are quite variable (3). The wide spectrum of functional perturbations induced by the different HCM mutations suggests that many different pathways lead to the HCM phenotype, so it is difficult to establish a prognosis based on the mutation (4). Dilated cardiomyopathy is the leading cause of heart transplantation and is mainly characterized by cardiac hypocontractility and enlargement of the ventricular chambers (5, 6). In both hypertrophic and dilated cardiomyopathy, these pathological phenotypes can lead to progressive heart failure (HF) and in some cases sudden cardiac death.

The primary cause of HCM is most often a mutation in one of several sarcomeric proteins, including cMyBP-C, myosin, and actin. cMyBP-C modulates muscle contraction and relaxation by interacting with both thin (actin-based) and thick (myosin-based) filaments (Fig. 1*A*). Numerous studies have demonstrated that increasing or decreasing protein kinase A (PKA)-mediated phosphorylation of cMyBP-C allows for tuning cardiac contraction and relaxation through phosphorylation-sensitive interactions with the myofilaments. Recent work by us (7) and others (8) suggests that phosphorylation induces a structural rearrangement in the N-terminal domains of cMyBP-C, which reduces its binding to actin and myosin, affecting muscle contraction and relaxation. Decreases in phosphorylation of cMyBP-C have been observed in patients with HF, including those affected by mutations in sarcomeric proteins other than cMyBP-C (9, 10). Therefore, targeting cMyBP-C with drugs that mimic phosphorylation and/or perturb its interactions with actin or myosin is a promising approach to improving cardiac muscle function in HF and cardiomyopathy.

**Figure 1:**
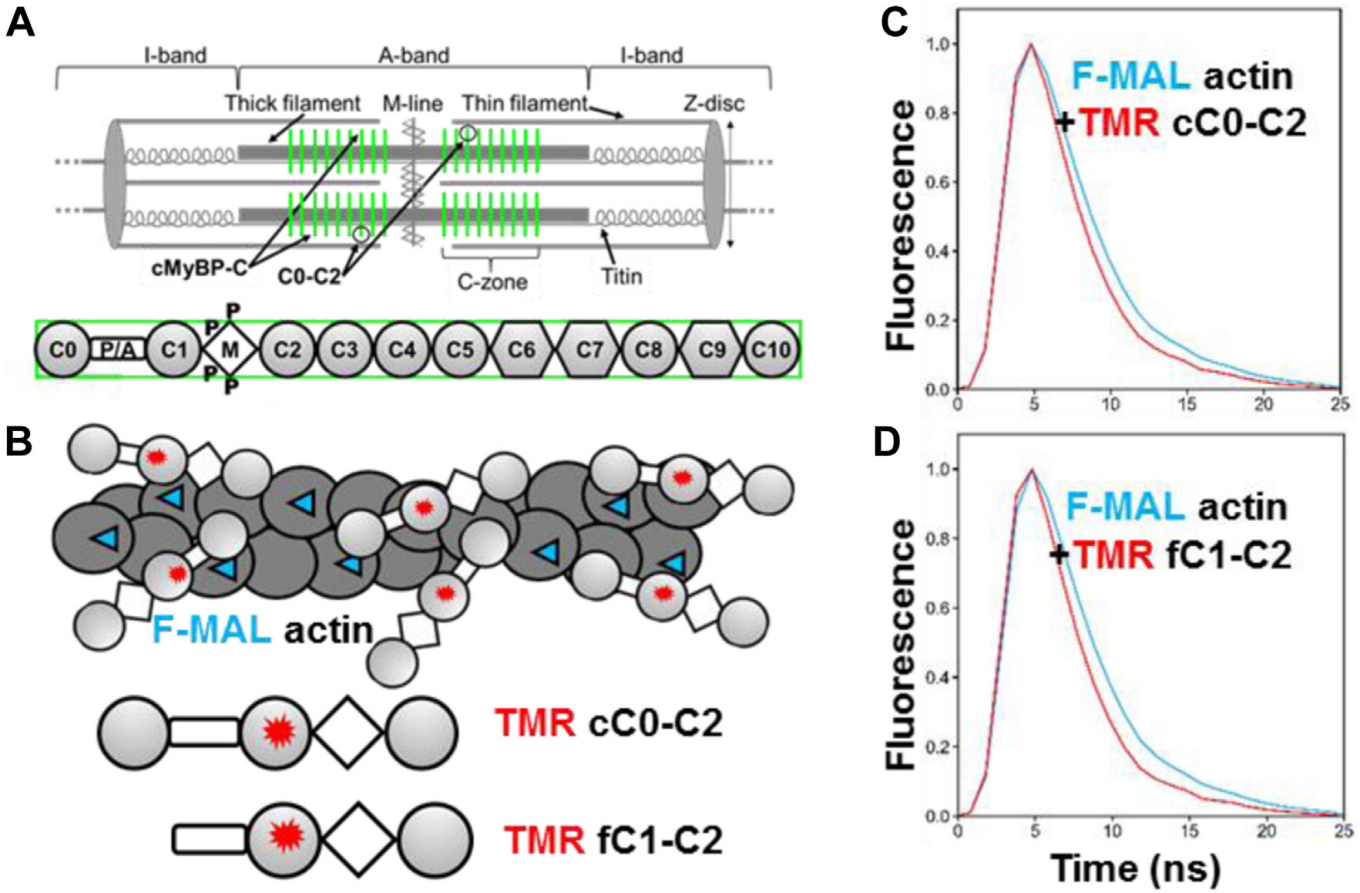
**cMyBP-C organization in the sarcomere.***A*, *top*, the sarcomere spans from Z-disc to Z-disc, with the A-band containing thick filaments and the I-band containing actin filaments. Force is generated by myosin and actin in the thin/thick filament overlap portion of the A-band. cMyBP-C molecules (*green vertical stripes*) are anchored to the thick filament and present in the C-zones toward the center of the A-band. The C-zones overlap with thin filaments (except at very long sarcomere lengths). Adapted from (21). *Bottom*: Full-length cMyBP-C domains C0 through C10. Ig-like domains are shown as *circles* and fibronectin type-III domains are shown as hexagons. N-terminal domains C0 through C2 (C0-C2), contain the flexible proline alanine-rich linker (P/A) and the partially disordered M-domain (M) that contains phosphorylation sites (P). Skeletal MyBP-C does not have C0. *B*, FMAL-actin and TMR-cC0-C2/fC1-C2 biosensor. The TMR label is located in the C1 domain. *C* and *D*, fluorescence waveform of FMAL-actin shows a faster decay in the presence of acceptor-labeled protein (*red curve*) than in the absence (*blue curve*), indicating FRET. Adapted from (13).

Small-molecule modulators targeting sarcomeric proteins have been developed recently, with a few progressing to clinical trials. Omecamtiv mecarbil, a selective cardiac myosin activator (11) (developed by Cytokinetics, Inc), and mavacamten, a selective β-cardiac myosin inhibitor (developed by MyoKardia, Inc) (12), are so far the most promising examples. However, hypertrophic and dilated cardiomyopathies as well as other types of HF are diverse in their symptoms and remodeling, so a variety of modulators, targeting other sarcomeric proteins, is desirable in order to provide adequate treatment options for patient populations.

We previously reported a high-throughput screen (HTS) assay, based on the detection of fluorescence lifetime (FLT) with sub-nanosecond (ns) time resolution, and identified three unique cMyBP-C-binding compounds (13). However, these compounds were subsequently found to not be specific for cMyBP-C, as they inhibit fast skeletal MyBP-C (fMyBP-C) interactions with actin (Fig. S1) and bind to both slow skeletal and fast MyBP-C N-terminal domains (Fig. S2). In the current study, we have extended and enhanced our previous approach, developing and performing two complementary high-throughput FLT-based screens, followed by isoform-specific secondary assays, to identify compounds that are muscle-type specific. We labeled F-actin with fluorescein-5-maleimide (FMAL), and the N-terminal fragment of cMyBP-C (cC0-C2) with tetramethylrhodamine (TMR). We first performed HTS to identify compounds that influence the interaction between actin and cC0-C2, using FLT-detected fluorescence resonance energy transfer (FLT-FRET) from donor-labeled actin to acceptor-labeled cC0-C2. Donor FMAL-actin was excited at 473 nm, and FLT-FRET to acceptor TMR-cC0-C2 was measured using a high-precision, high-speed fluorescence lifetime plate reader (14). We performed a second screen, measuring FLT of TMR-cC0-C2, using the same samples in the same plate, to identify compounds that bind directly to cC0-C2, whether or not they affect its actin binding. Here, TMR-cC0-C2 was excited at 532 nm using a different laser in the fluorescence lifetime plate reader, and FLT changes due to the presence of compound identified cC0-C2-binding structural modulators. These compounds are also potential effectors for cC0-C2 interactions with myosin. Using these two approaches, we screened a 2684-compound Selleck library of FDA-approved compounds, with the goal of identifying cMyBP-C-binding compounds that modulate its functional activities in the sarcomere.

We established additional and reliable biochemical and structural secondary assays, which had lower throughput than the primary FLT assays, but were rapid and precise enough to test whether the identified initial “hit” compounds from the primary HTS screens are specific for cMyBP-C *versus* fMyBP-C. Differences in binding to phosphorylated *versus* non-phosphorylated cC0-C2 were also measured. Finally, we tested the effects of these compounds on the ATPase activities of skeletal and cardiac myofibrils to determine whether these hits modulate muscle type-specific function. This process to identify and develop lead compounds targeting cMyBP-C is summarized in Figure 2.

**Figure 2:**
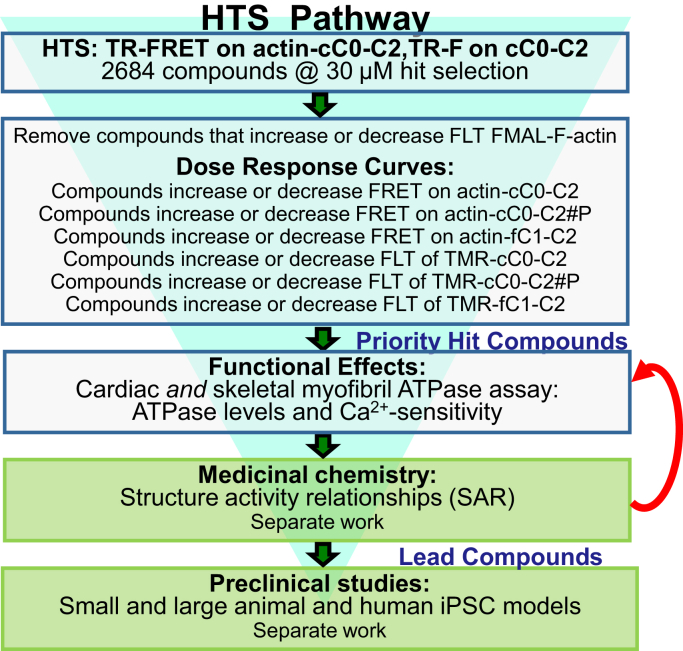
**High-throughputscreening process (funnel) to find compounds targeting cMyBP-C.** Top three *clear boxes* summarize the experimental design of the present study. Bottom two *green boxes* highlight future efforts in separate work.

The success of these high-throughput primary and secondary assays, demonstrated below, gives us confidence that this approach can be expanded to much larger libraries to identify more compounds to serve as starting points for the medicinal chemistry that will probably be necessary to increase specificity and drug potency for the treatment of specific types of HF and cardiomyopathy.

## Results

### FLT-FRET of actin-cC0-C2/fC1-C2 biosensor

**Figure 3:**
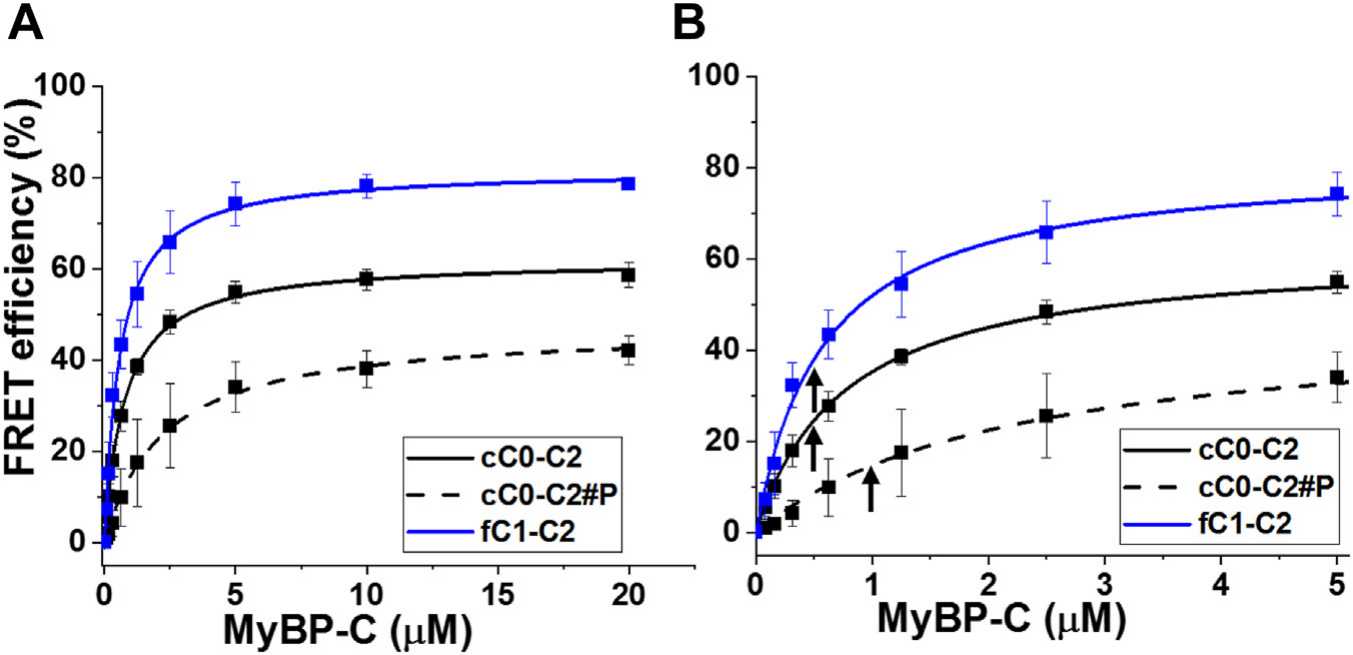
**Actin-cMyBPC FLT-FRET assay.***A*, FLT-FRET-based binding curve of 0.25 μM FMAL-actin and 0 to 20 μM TMR-cC0-C2 (*solid black line*, unphosphorylated), PKA-phosphorylated (#P) TMR-cC0-C2 (*dashed black line*) and TMR-fC1-C2 (*solid blue line*). Phosphorylation of cC0-C2 decreases its binding to actin. *B*, expansion of 0 to 5 μM of (*A*). The concentration of TMR-cC0-C2 and TMR-fC1-C2 (0.5 μM) and phosphorylated TMR-cC0-C2 (1 μM) used in the HTS screen and in testing of compound effects is indicated with arrows. Data are mean ± SD. n = 7 to 10 from N = 2 separate actin and cC0-C2 or fC1-C2 preparations. All values are the average ± SD.

Using an FLT-detected cC0-C2-actin binding assay, we previously identified the first three compounds that bind to cC0-C2 and inhibit its interactions with actin (13, 15). However, these compounds were subsequently found to not be specific for cMyBP-C, as they inhibit fC1-C2 interactions with actin (Fig. S1). The effects of these compounds on cC0-C2 binding were confirmed with a novel FLT-based FRET assay, in which donor FMAL-actin and acceptor TMR-cC0-C2 are mixed. Binding is indicated by a decrease in FLT (increase in FRET) of FMAL-actin due to the close proximity (binding) of TMR-cC0C2. The FRET response (∼45% decrease in FLT) was much larger than detected in the absence of acceptor with unlabeled cC0-C2 (∼7% decrease in FLT) (13) Therefore, we chose to monitor FLT-FRET in the current screen (Fig. 1, *B*–*D*). Preliminary trials, using the same concentration (1 μM) of FMAL labeled F-actin plus 2 μM of TMR-cC0-C2 resulted in clogging of the dispensing pins of the multidrop liquid dispenser. Therefore, we reduced the concentration of donor (FMAL-actin) and acceptor (TMR-cC0-C2) to 0.25 μM and 0.5 μM, respectively. FLT-FRET binding curves, using these concentrations of FMAL-actin and TMR-cC0-C2, indicated that these concentrations are suitable to use in screening for compounds that decrease or increase binding (Fig. 3). Binding curves for PKA-phosphorylated TMR-cC0-C2 (cC0-C2#P) and TMR-fC1-C2 were generated, to be used in secondary assays, to identify isoform- and phosphorylation-specific compounds (Fig. 3). These FLT-FRET binding curves were fit to a hyperbolic function and yielded K_d_ values of 0.76 ± 0.020 μM (cC0-C2), 2.21 ± 0.171 μM (protein kinase A-phosphorylated cC0-C2#P), and 0.58 ± 0.036 μM (fC1-C2).

### FLT of TMR-cC0-C2 biosensor

In addition to dramatically reducing the FLT-FRET between FMAL-actin and TMR-cC0-C2, the binding of previously identified compounds to labeled cC0-C2 changed the FLT of TMR-cC0-C2 during direct excitation (Fig. S2). This suggests that by using the same FLT-FRET samples containing labeled FMAL-actin and labeled TMR-cC0-C2, we could screen first for compounds that modulate cC0-C2 binding to actin by monitoring FMAL-actin FLT (excited at 473 nm) and then screen for compounds that bind to cC0-C2 by re-reading the plate and monitoring the FLT of TMR-cC0-C2 (excited at 532 nm). Thus, we were equipped to perform a dual screen to identify MyBP-C-specific compounds.

### High-throughput screening of an FDA-approved library for compounds modulating cC0-C2-actin interactions and compounds that bind cC0-C2

Using the FMAL-actin plus TMR-cC0-C2 FRET biosensor, we performed HTS of the Selleck library that contains 2684 FDA-approved compounds (drugs and active pharmaceutical ingredients). The entire screen was performed in duplicate with two different preparations of FMAL-actin and TMR-cC0-C2 samples. To ensure the suitability of these biosensor preparations for screening, we computed the assay Z′, a standard metric in high-throughput screening, to determine the sensitivity of an HTS assay for detecting novel effectors (see Experimental procedures, Equation 2). A Z′ value >0.5 indicates an assay of excellent quality that is ready for high-throughput screening (16). Z′ was calculated as 0.61 for the first screen and 0.63 for the second using DMSO-only controls comparing over 600 wells with and without TMR-cC0-C2 (Table S1). The quality of the HTS screens was also validated by calculating Z' (0.62 ± 0.01) using FMAL-actin plus TMR-cC0-C2 FLT-FRET without and with suramin (a compound identified in our earlier study) (13), which eliminates binding between actin and cC0-C2.

**Figure 4:**
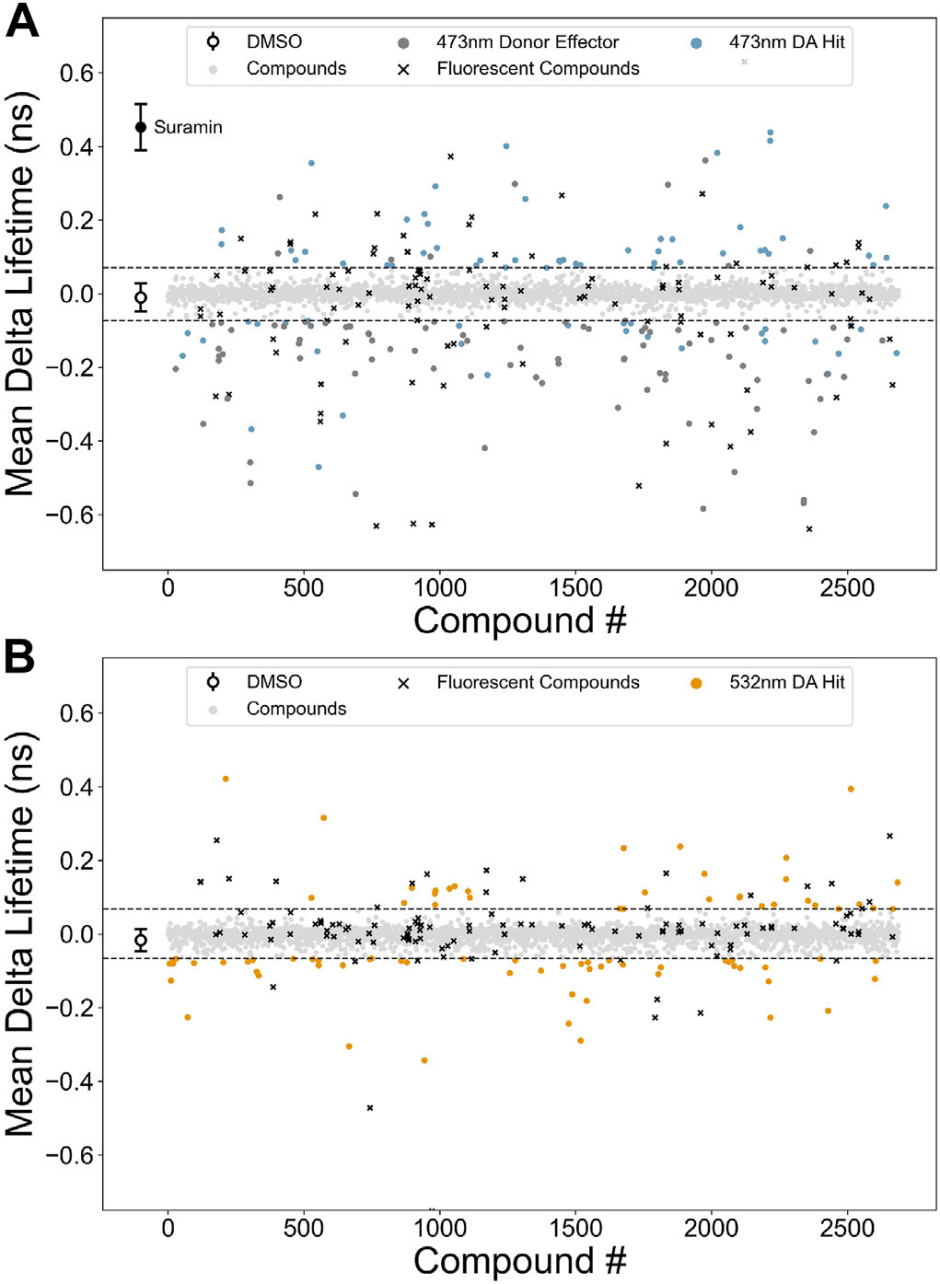
**A representative screen of the Selleck library.** Screening was done in duplicate with two different preparations of FMAL-actin and TMR-C0-C2 and reproducible hits were identified. Hits are identified as exceeding a z-score of ±4 of the plate-wide statistics. *A*, excitation at 473 nm. Reproducible donor-only (*dark gray dots*) and donor-acceptor (DA) (*blue*) hits at 473 nm. Donor-only hits are excluded from the ‘hits’ list as they affect the lifetime of FMAL-actin directly. Fluorescent compounds are shown in *black* and compounds that do not change FLT beyond the hit threshold are shown in *light grey*. *B*, excitation at 532 nm. Reproducible hits changing the FLT of TMR on cC0-C2 are shown as *orange dots*.

Each screen monitored the effect of the compounds (1) on the FLT of FMAL-actin (donor only) at 473 nm in the absence of acceptor (2), on the FLT of FMAL-actin in the presence of TMR-cC0-C2 (donor-acceptor) at 473 nm and (3) on the FLT of TMR-cC0-C2 (acceptor) in the presence of FMAL-actin at 532 nm (Fig. 4). The statistical distribution of compound-induced changes of FLT (compared to DMSO) was computed using the robust z-score (See Experimental procedures, Equation 4). Compounds with a z-score < −4 or > +4 were deemed sufficiently distant from normal biosensor fluctuation and therefore likely true positives warranting further consideration. Using these criteria, we selected 60 reproducible hit compounds (Table S2) that either decreased (Table 1 and Fig. 4*A*) or increased (Table 2 and Fig. 4*A*) FRET between FMAL-actin and TMR-cC0-C2, and/or bound to TMR-cC0-C2 causing its FLT to decrease or increase (Table 3 and Fig. 4*B*). Compounds that influence FRET between FMAL-actin and TMR-cC0-C2 are potential effectors for actin-cMyBP-C interactions, whereas compounds that impact FLT of TMR-cC0-C2 bind to cC0-C2 and likely affect its structure. Those not affecting actin binding (underlined in Table 3) have the potential to modulate interactions with myosin. 60 reproducible hits were selected to study concentration–response effects.

**Table 1:** Compounds decreasing FMAL-actin- TMR-cC0-C2 FLT-FRET

FLT-FRET change at 60 μM compound concentration is shown, except ∗ (10 μM). Effects (% change from DMSO) on FLT-FRET between FMAL-actin and TMR-MyBP-C (FMAL FLT-FRET). Red indicates a 5-fold greater effect on cC0-C2 compared to phosphorylated cC0-C2 and green indicates 5-fold greater effect on cC0-C2 compared to fC1-C2.

**Table 2:** Compounds increasing FMAL-F-actin- TMR-C0-C2 FRET

FRET change at 60 μM compound concentration is shown. Effects (% change from DMSO) on FLT-FRET between FMAL-actin and TMR-MyBP-C (FMAL FLT-FRET). Red indicates a 5-fold greater effect on cC0-C2 compared to phosphorylated cC0-C2. Green indicates a 5-fold greater effect on cC0-C2 compared to fC1-C2.

**Table 3:** Compounds Affecting TMR-FLT of cC0-C2, cC0-C2#P and fC1-C2

Compounds	cC0-C2	cC0-C2#P	fC1-C2		
	FLT change	FLT change	cC0-C2/cC0-C2#P	FLT change	cC0-C2/fC1-C2
Erythromycin estolate	−14.1%	−2.4%	5.9	−2.1%	6.7
Clindamycin palmitate HCl	−12.7%	−10.2%	1.2	−1.2%	10.6
Scutellarin	−9.1%	−8.7%	1.0	−9.5%	1.0
Fidaxomicin	−5.2%	−0.5%	10.4	−4.3%	1.2
Fenticonazole nitrate	−4.7%	−6.0%	0.8	0.4%	11.8
Bedaquiline fumarate	−4.5%	−6.5%	0.7	6.7%	0.7
Fostamatinib (R788)	2.3%	0.7%	3.3	0.4%	5.8
Chloroquine phosphate	2.5%	3.1%	0.8	1.9%	1.3
Hederagenin	2.5%	3.6%	0.7	2.3%	1.1
Enoxaparin sodium	3.1%	6.9%	0.4	2.8%	1.1
Tocofersolan	3.3%	3.2%	1.0	2.8%	1.2
Tamibarotene	5.2%	7.2%	0.7	5.9%	0.9
Saikosaponin D	5.4%	6.3%	0.9	4.2%	1.3
Verteporfin∗	5.5%	6.8%	0.8	2.5%	2.2
Troxerutin	6.1%	7.3%	0.8	6.0%	1.0
α-Hederin	6.7%	7.1%	0.9	6.7%	1.0
Cefsulodin sodium	9.1%	8.1%	1.1	11.5%	0.8
Febuxostat	9.6%	12.1%	0.8	14.6%	0.7
Erlotinib	9.8%	9.7%	1.0	8.5%	1.2
Nilotinib hydrochloride	10.1%	14.1%	0.7	15.6%	0.6
Ertapenem sodium	10.2%	8.0%	1.3	12.2%	0.8
Triclocarban	11.6%	18.2%	0.6	22.2%	0.5
Erlotinib HCl	11.8%	13.9%	0.8	11.1%	1.1
Pneumocandin B0	12.4%	13.2%	0.9	11.4%	1.1
Paritaprevir (ABT-450)	23.8%	20.3%	1.2	11.0%	2.2
Pranlukast	30.7%	50.1%	0.6	50.3%	0.6
Anidulafungin	32.1%	43.3%	0.7	53.0%	0.6

FLT change at 60 μM compound concentration is shown, except ∗ (10 μM). Effect (% change from DMSO) on FLT of TMR-MyBP-C. Compounds that did not affect cC0-C2 or fC1-C2 binding to actin (not in Tables 1 and 2) are underlined.

### Concentration–response curves

Compounds identified as initial hits were further examined to determine the concentration–response curves for seven conditions (1): FMAL-actin alone identified actin-binding compounds that alter the FLT of FMAL-actin (2). FMAL-actin plus TMR-cC0-C2, focusing on a change in FLT of FMAL due to FRET, as in the initial screen, monitored compound effects on the interaction of actin with cC0-C2 (3). FMAL-actin plus PKA-phosphorylated TMR-cC0-C2, focusing on a change in the FLT of FMAL (FRET), monitored compound effects on the interaction of actin with phosphorylated cC0-C2 (4). FMAL-actin plus TMR-fC1-C2, focusing on the FLT of FMAL (FLT-FRET), monitored compound effects on the binding of actin with fC1-C2 (5). TMR-cC0-C2, focusing on the FLT of TMR, monitored cC0-C2-binding compounds (6). Phosphorylated TMR-cC0-C2, focusing on the FLT of TMR, monitored phosphorylated cC0-C2-binding compounds (7). TMR-fC1-C2, focusing on the FLT of TMR, monitored fC1-C2-binding compounds. Of the 60 initial hit compounds chosen for concentration–response curves, 22 either increased the donor-only lifetime at the higher compound concentration or showed very little effect on concentration–response curves. Concentration–response curves for the remaining 38 compounds are summarized in Table 1, Table 2, Table 3 with their effects at 60 μM compound concentration.

In the concentration–response curves, 23 compounds decreased FRET between actin and cC0-C2 (Table 1). Valsartan, Sacubitril/Valsartan (LCZ696), Clinofibrate, Erlotinib, Zaltoprofen, Laquinimod, and Erythromycin estolate, showed specificity for cC0-C2 as the effect on fC1-C2 FRET (compared to cC0-C2) was reduced greater than fivefold. These same compounds showed a stronger reduction of FRET with non-phosphorylated than phosphorylated cC0-C2. Twelve of the 23 compounds that decreased FRET also showed changes in TMR FLT, indicating that they bind directly to MyBP-C (compare Tables 1 and 3).

Eight of the nine compounds identified as increasing FRET, between FMAL-actin and TMR-cC0-C2, altered the FLT of TMR on cC0-C2 and/or fC1-C2, indicating that they bind to MyBP-C (compare Tables 2 and 3). Verteporfin displayed the greatest specificity for cC0-C2 over fC1-C2 (22.4-fold). Saikosaponin D, α-Hederin, and Tocofersolan showed the largest specificity for unphosphorylated over phosphorylated cC0-C2 (13–54-fold). In contrast, Fenticonozole nitrate showed the strongest preference for increasing the binding of the phosphorylated over unphosphorylated cC0-C2 (5-fold). Interestingly, Pneumocandin B0 increased the binding of unphosphorylated cC0-C2 and fC1-C2 while decreasing the binding of phosphorylated cC0-C2. Tocofersolan affected the binding to actin of unphosphorylated cC0-C2 and fC1-C2, with no effect on phosphorylated cC0-C2, despite binding to it (see changes in TMR FLT, Table 3) (Table 2).

Of the 27 compounds that changed the FLT of unphosphorylated TMR-cC0-C2, 23 also changed the FLT of phosphorylated TMR-cC0-C2 and TMR-fC1-C2 to similar extents (0.6–2.2-fold differences). Larger differences in the effects on TMR FLT, indicative of selective binding, between unphosphorylated and phosphorylated TMR-cC0-C2, were seen with Erythromycin estolate (5.9-fold), Fidaxomicin (10.4-fold) and Fostamatinib (R788) (3.3-fold). Similar indications of selective binding between unphosphorylated TMR-cC0-C2 and TMR-fC1-C2 were observed for Erythromycin estolate (6.7-fold), Clindamycin palmitate (10.6-fold), Fenticonazole nitrate (11.8-fold) and Fostamatinib (R788) (5.8-fold) (Table 3). Two other compounds, 8-Hydroxyquinoline and Benzonate, strongly reduced the FLT of TMR-cC0-C2 in both replicates of the initial screen (Table S2) but did not in the concentration–response curves. This may indicate that the reduction in FLT was dependent on the presence of actin (present in the initial screen), which was absent in the concentration–response curve test for MyBP-C binding.

**Figure 5:**
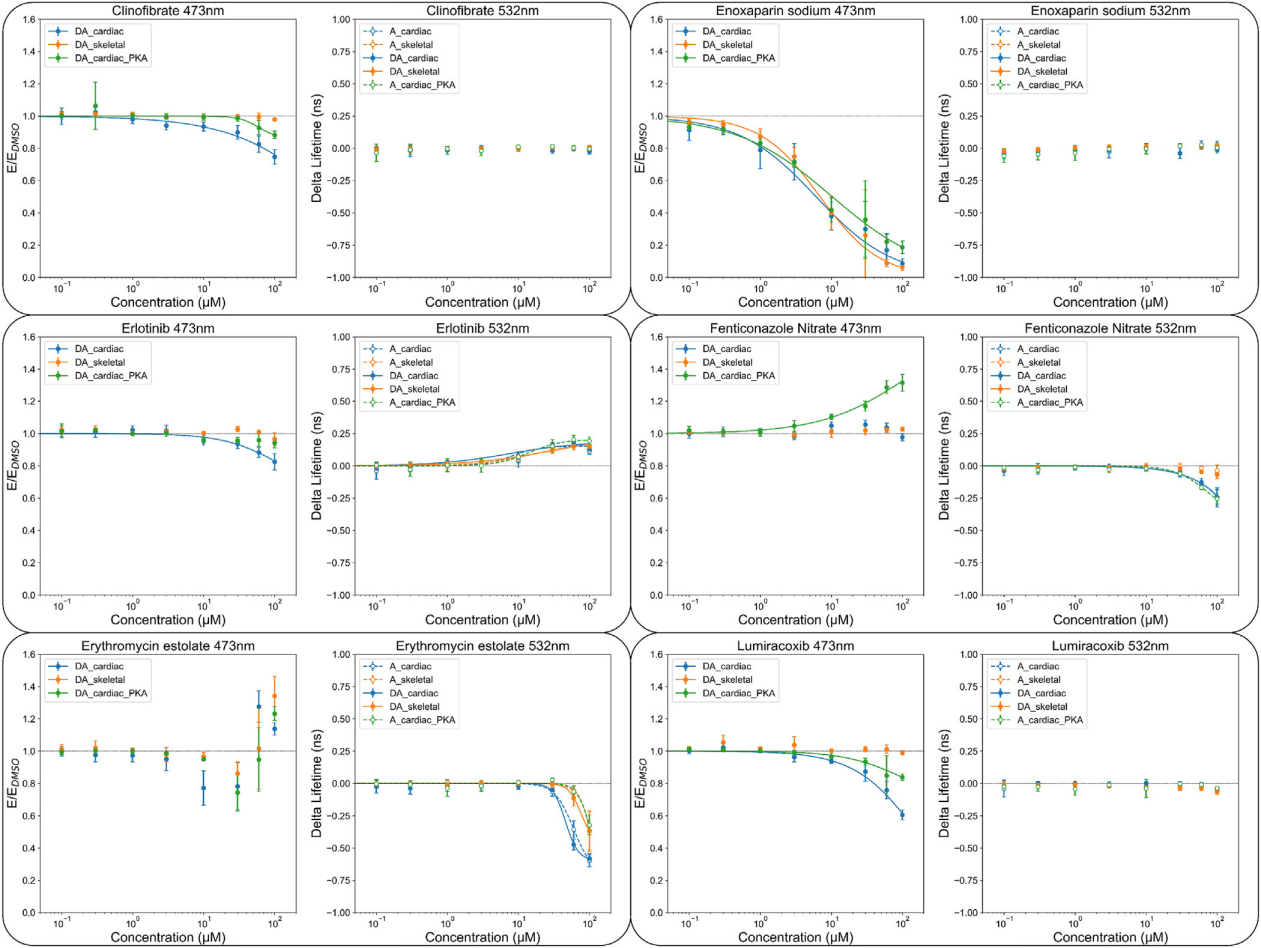
**Concentration-response curves of representative hits.** Samples are excited at 473 nm or 532 nm, with the 473 nm data plotted as normalized FRET efficiency (E/E_DMSO_) and the 532 nm data plotted as the change in the acceptor lifetime compared to DMSO (Delta lifetime). To help guide the eye, data were fit to a hill equation when the data exceeded a 10% change from baseline and was not biphasic. Clinofibrate, Enoxaparin sodium, Erlotinib, Lumiracoxib decrease the FLT-FRET between FMAL-actin and TMR-cC0-C2 (donor-acceptor (DA)_cardiac). Fenticonazole nitrate increases FLT-FRET between FMAL-actin and PKA-phosphorylated TMR-cC0-C2 (DA_cardiac_PKA) but does not have any effect on cC0-C2 (DA_cardiac) or fC1-C2 (DA_skeletal). Erthromycin estolate has a biphasic effect on FRET between FMAL-actin and TMR-cC0-C2/PKA-phosphorylated-TMR-cC0-C2/TMR-fC1-C2. Erlotinib increases TMR-cC0-C2 FLT for all samples, and Erthromycin estolate decreases them. Fenticonazole nitrate slightly decreases FLT of TMR-cC0-C2 (acceptor (A)_cardiac) and PKA-phosphorylated-TMR-cC0-C2 (A_cardiac_PKA) but does not affect TMR-fC1-C2 (A_skeletal). Data are collected from three independent preparations of actin and TMR-cC0-C2 and TMR-fC1-C2. N = 3, n = 9. The effects on FLT-FRET and FLT values at 60 μM compound concentration are summarized in Table 1, Table 2, Table 3. All values are in average ± SD.

These secondary assays have allowed us to categorize effectors from the screens that modulate either the structural complex of actin-cC0-C2 and/or bind to cC0-C2 with varying degrees of specificity. Complete concentration–response curves are displayed for representative compounds of different categories (Fig. 5).

### Cardiac and skeletal muscle ATPase assays

A set of selected compounds that showed significant effects on concentration–response curve structural assays and/or specificity toward skeletal or cardiac MyBP-C fragments were further tested for their effects on muscle function. Compound effects were examined on the Ca^2+^-dependent ATPase activity of bovine cardiac (left ventricular) myofibrils (cardiac MF) and rabbit fast skeletal (psoas) myofibrils (skeletal MF) in a moderate-throughput manner (*i.e.*, ∼30 min assay 96-well plates), varying the free calcium concentration from pCa 8 (relaxation) to pCa 4 (full activation). The effects of 22 current hits and two previously identified compounds (NF023 and suramin) (13) were tested for their effects on the myosin ATPase activity in cardiac and skeletal MF. The pCa_50_ values of cardiac and skeletal MF are 5.77 ± 0.03 and 6.07 ± 0.02, respectively, consistent with previous reports (17).

**Figure 6:**
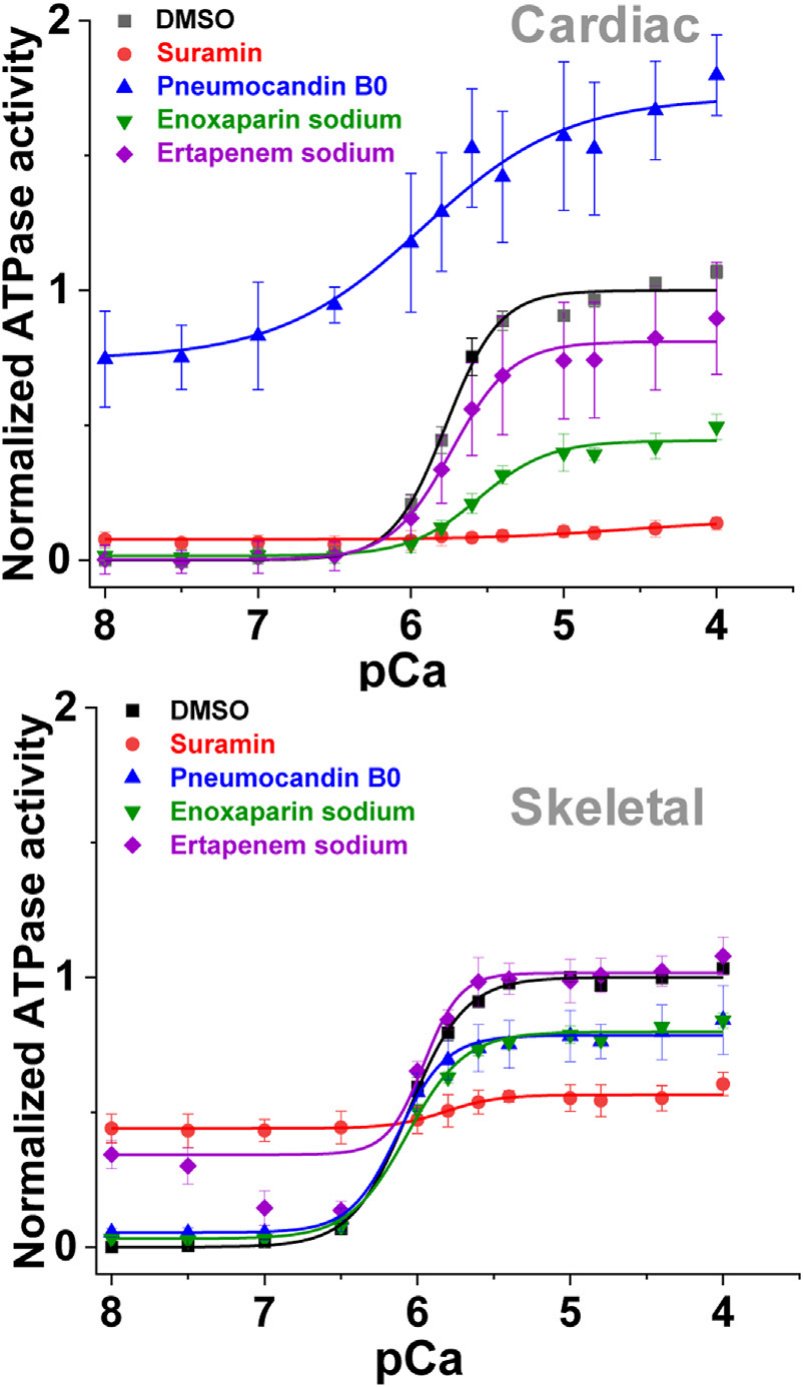
**Myofibril ATPase assay.** ATPase activity of bovine cardiac myofibrils (*top*) and rabbit skeletal myofibrils (*bottom*) were measured in the presence of 1% DMSO and 50 μM compound across a 12-point Ca^2+^ gradient, pCa 4 to 8, in a 96-well–plate. Error bars are SD (N = 3, n = 6 at each Ca^2+^ concentration). ATPase activity was calculated as a micromole of ATP/mg of protein/min scale. Graphs are normalized to the DMSO-only sample for better visualization of the compound effect on cardiac and skeletal. Calculated ATPase rates at pCa 4 and at pCa 8 are summarized in Table 4.

The effects of the representative compounds on myofibril (MF) ATPase (Table 4 and Fig. 6) varied among compounds. Suramin eliminated the Ca^2+^-sensitivity of ATPase in both cardiac and skeletal MF. Cardiac MF ATPase activities remained at levels similar to those at low Ca^2+^, while the skeletal MF ATPase levels showed activation at low Ca^2+.^ The degree of inhibition at higher Ca^2+^ is also different (Fig. 6). Pneumocandin B0, showed an ATPase-activating effect for cardiac MF at low and high Ca^2+^ and an inhibitory effect for skeletal MF at high Ca^2+^. Enoxaparin sodium showed a significant decrease in Ca^2+^-sensitivity (pCa 5.55 ± 0.03 *versus* 5.77 ± 0.03 in the absence of the drug) for cardiac MF, whereas its effect on skeletal MF was negligible. In both cardiac and skeletal MF, ATPase levels were reduced at high Ca^2+^. Ertapenem sodium showed altered Ca^2+^ sensitivity for the skeletal MF, and a marked increase in ATPase activity at pCa 8 for skeletal MF. In addition, Pranlukast, Erlotinib, Cefsoludin sodium, and Cefepime dihydrochloride monohydrate affected skeletal MF ATPase activity, and NF023 affected cardiac MF ATPase activity and Ca^2+^-sensitivity (Table 4). These ATPase assays in whole myofibrils validate our myofilament protein-level HTS screening platform’s ability to identify compounds with functional significance.

**Table 4:** Myofibril ATPase activity

ATPase is calculated as micromol ATP/min/mg of myofibril. Compound concentration was 50 μM, except ∗ (10 μM). All values are the average ± SD. N = 3 (n = 6 for each Ca^2+^ concentration). Compounds in **bold** are shown in Figure 6 and significant differences in ATPase compared to DMSO are shown in red.

## Discussion

We report a novel approach to discover two distinct classes of compounds that affect the binding of the N-terminal cC0-C2 fragment of cMyBP-C to actin and/or myosin, with the aim of finding improved therapies for HF and cardiomyopathy patients. Here, we have developed two parallel HTS screens, performed with the same microplate, that successfully identified compounds with the potential to influence muscle regulation by binding to MyBP-C.

The ultimate goal of our work is to identify novel small-molecule effectors with therapeutic potential for cardiac disorders associated with hypo- or hyper-contractility and diastolic and/or systolic dysfunction. This includes cardiomyopathy (*e.g.*, dilated cardiomyopathy or hypertrophic cardiomyopathy) and HF (*e.g.*, HF with preserved or reduced ejection fraction). Small-molecule effectors designed to target striated muscle proteins, such as myosin and troponin, for the treatment of disease are showing promise in preclinical and clinical trials (12, 18). In our work, we focus on the modulation of cMyBP-C interactions with both actin and myosin. This approach is novel, as targeting cMyBP-C offers many of the same advantages for modulating heart function as targeting myosin or actin directly. One benefit of cMyBP-C being a cardiac-specific accessory protein is that it increases the opportunity to discover cardiac-specific drug candidates. Thus, it is likely that drugs binding to cMyBP-C will have reduced nonspecific side effects.

Data from the current FRET biosensor (FMAL donor on actin and TMR acceptor on cC0-C2) is sensitive enough to detect (with high *precision*) very small (sub-nanometer level) *changes* in the structure of the protein complex due to the addition of a compound, as needed for screening. The FLT-FRET that we observe between FMAL-actin and TMR-cC0-C2 is consistent with the distances observed by others (19, 20). However, due to multiple factors, this data is not sufficient to determine *absolute* distances *accurately*. The distances from one actin at Cys-374 to C1 (in cC0-C2) at Cys-249 are either 3.14, 5.23, 6.68, or 6.98 nm depending on whether the C1 domain is bound to the labeled actin monomer or neighboring actin monomers (from PDB 6CXI) (20). The FMAL-TMR FRET pair has an R_0_ of 5.5 to 5.9 nm and FRET is detected when the probes are spaced by less than 8.9 nm. This means that all four of these binding sites (donor-acceptor distances) would contribute to FRET. Also, when multiple C0-C2 molecules are bound in this region there will be contributions from multiple acceptor probes. This is potentially further complicated by the multiple modes of binding (or binding sites) of cC0-C2 on each actin monomer (19, 20, 21).

Consistent with earlier results by us (15, 22) and others (23), the FRET assay used here detects actin-cMyBP-C N-terminal binding in concentration- and phosphorylation-dependent manners (Fig. 3). The various binding assays (FRET, FLT, and cosedimentation) were each fit to a hyperbolic function yielding apparent affinities and K_d_ values, and treatment with PKA to phosphorylate cC0-C2 reduces binding. K_d_ values for the current FRET assay, using 0.25 μM FMAL-actin and TMR-cC0-C2 (0.8 and 2.2 μM for cC0-C2 and phosphorylated cC0-C2, respectively) are lower than that observed with higher concentrations of actin. We previously observed K_d_ values of 4.3 μM for cC0-C2 and 6.5 μM for PKA-phosphorylated-cC0-C2 in a cosedimentation assay, and 2.5 and 19 μM for cC0-C2 and PKA-phosphorylated cC0-C2 in an FLT assay using 1 μM actin (15). These differences are probably due to C0-C2’s capacity for multiple modes of binding (or binding sites) on each actin monomer (19, 20, 21). For this reason, the apparent K_d_ values for cC0-C2 binding to F-actin should be viewed with caution (15). Despite these complications, the binding curves clearly indicated decreased binding of PKA-treated cC0-C2, and they identified suitable concentrations for the HTS screening assays (using cC0-C2, phosphorylated cC0-C2, and fC1-C2) that resulted in significant, but submaximal FRET values (arrows in Fig. 3*B*). At submaximal binding levels, we were able to identify compounds that either decreased or increased the interactions between cC0-C2 (and fC1-C2) in our FRET-based assays.

Compounds identified in our HTS screens that alter the interaction between F-actin and cC0-C2 or bind to cC0-C2 were readily detected from a library of 2684 FDA-approved compounds. The optimized HTS assay using FMAL-actin and TMR-cC0-C2 showed Z′ values of 0.61 and 0.63 in two tests in the 1536-well plate format (Table S1). Therefore, our primary screen is considered excellent in the field of HTS assay development (16).

Sub-goals of this work were (1) to establish an HTS screening platform to identify compounds that specifically bind to cardiac MyBP-C and potentially (2) to identify specific compounds that regulate cardiac contractile functions, such as myosin ATPase. Our secondary assays on 60 reproducible hits from the primary FLT-FRET and FLT-TMR assays did identify compounds showing specificity of effects on cardiac *versus* skeletal MyBP-C, on unphosphorylated *versus* phosphorylated MyBP-C, and on donor actin. Thus, our first sub-goal was achieved. For our second sub-goal, one of these compounds (Enoxaparin sodium) specifically altered the Ca^2+^-sensitivity of cardiac myosin ATPase.

The interpretations of FLT-FRET and FLT-TMR values are different, providing complementary information. For FLT-FRET, the magnitude and direction of FLT-FRET changes correlate with cC0-C2 or fC1-C2 interactions with actin. An increase (or decrease) in FLT-FRET indicates, quantitatively, an increase (or decrease) in protein–protein binding. For FLT-TMR, the structural effects are less clear, indicating only that a compound binds and alters the environment of the protein-bound TMR, without a quantitative measure of the effect on protein structure or binding. The lack of a change in FLT-TMR does not imply a lack of binding. Compounds can bind to cC0-C2 or fC1-C2 without affecting the lifetime of TMR. The same is true for the magnitude of FLT changes of FMAL-actin in the absence of cC0-C2 or fC1-C2.

As HCM and HF often result in hypophosphorylation of MyBP-C and are presumably associated with increased actin binding, we focus primarily on the first group of compounds that decrease actin binding, assessed by decreased FRET. We are particularly interested in those compounds reducing FMAL-actin/TMR-cC0-C2 FRET specifically, or preferentially, as they may mimic the effects of phosphorylation on the binding of cC0-C2 to actin. Looking at specificity indicators (differences in the change in FRET efficiency for unphosphorylated cC0-C2 compared with phosphorylated cC0-C2 or fC1-C2) in Table 1, we find four compounds (Elbasavir, Fostamatininb [R788], Erythromycin estolate and Erlotinib) that show a 5-fold greater effect with unphosphorylated cC0-C2 than phosphorylated (these specificities are indicated in red in Table 1). Seven compounds (Sacubitril/Valsartan (LCZ696), Valsartan, Zaltoprofen, Laquinimod, Erythromycin estolate, Clinofibrate and Erlotinib) show specificity toward cC0-C2 (5-fold or greater effect, indicated by green in Table 1) over fC1-C2. Erythromycin estolate and Erlotinib overlap in these two categories. Five of these compounds (Elbasvir Sacubitril/Valsartan [LCZ696], Zaltoprofen, Laquinimod, and Clinofibrate) do not affect the lifetime of TMR on cC0-C2 or fC1-C2, suggesting that they do not bind to (and change the structure of) the C1 domain where TMR is located. Unphosphorylated *versus* phosphorylated cC0-C2 effects suggest that these five compounds bind to the M-domain only when it is unphosphorylated. Erythromycin estolate, Erlotinib and Fostamanitib (R788) also affect the TMR-FLT of cC0-C2. This suggests that binding of these compounds to cC0-C2 affects FLT of TMR on C1, and phosphorylation of the M-domain prevents or alters this interaction. Elbasavir, Fostamatinib (R788), Erythromycin estolate, Sacubitril/Valsartan (LCZ696), Valsartan, Zaltoprofen, Laquinimod, and Clinofibrate decreased the actin-binding of unphosphorylated cC0-C2, phosphorylated cC0-C2 and fC1-C2, but to different extents. Thus, these compounds exhibit isoform specificity and are worthy of further study.

A second group of compounds (Table 2) increased FRET between actin and cC0-C2, indicating increased binding. Four compounds (α-Hederin, Tocoforsolan, Saikosaponin, and Verteporfin, Table 2), showed a fivefold or greater effect on increased FRET (or binding). This is interesting, as we did not expect to find this group of compounds with our biosensor. These compounds could be useful for treating other cardiomyopathy or HF conditions.

Compounds that showed cC0-C2 specificity on FRET or FLT did not generally show specificity for cardiac myofibril ATPase (Table 4). Significant differences in ATPase rates at pCa 4 and pCa 8 as well as pCa_50_ compared to DMSO were observed (Table 4). Suramin (identified previously) (13) had the strongest effect. Cefepime dihydrochloride monohydrate, Cefsulodin sodium, Ertapenem sodium, Pranleukast, and Pneumocandin B0 affected ATPase in skeletal myofibrils, whereas Enoxaparin sodium, was specific for cardiac myofibril ATPase effects. All of these compounds were among the strongest in their effects on actin binding, reducing or increasing FRET by 40 to 99% in unphosphorylated cC0-C2 (53–90% in phosphorylated cC0-C2, and 34–96% in fC1-C2) and all displayed binding to TMR-C1 in all three proteins. Cefepime dihydrochloride monohydrate affected skeletal myofibril ATPase while having reduced effects on actin binding (13–35%) and showed no effect on the TMR-C1 in all three proteins. This suggests that there is not a strong correlation between actin-binding effects and modulation of myosin ATPase in cardiac myofibrils. This may indicate that compounds capable of regulating cardiac ATPase will need to alter MyBP-C interactions with myosin (separately or in addition to actin). The finding that compounds specific for unphosphorylated cC0-C2 did not affect cardiac myofibril ATPase activities or calcium sensitivity (change in pCa_50_) may be due to MyBP-C being phosphorylated (24) in the cardiac myofibrils used for our ATPase activities. The results may be different when looking for effects in the hypophosphorylated myocardium seen in HCM and HF (9). For skeletal MyBP-C, actin binding effects may be somewhat more predictive of effects on myofibril ATPase as, of the top five compounds affecting fC1-C2-actin FRET, three did modulate skeletal ATPase. This link is limited, however, exemplified by Erlotinib (no effect on actin binding), Cefepime Dihydrochloride Monohydrate (reduction of 13% in actin binding), and Pneumocandin B0 (increase of 24% in actin binding) that all affect skeletal muscle ATPase. Compounds that did not show any effect on actin-binding but did change the TMR-FLT of cC0-C2 could potentially affect the myosin binding of cC0-C2 (six, underlined in Table 3). One of these compounds, Erlotinib, affected skeletal myofibril ATPase.

In summary, our screening platform identified compounds that bind to and affect cC0-C2. It also identifies compounds that bind to phosphorylated cC0-C2 and fC1-C2. To achieve our end goal of identifying specific compounds that regulate cardiac contractile functions, like myosin ATPase, we anticipate using this platform to screen larger libraries, as we have done for effectors of myosin activity (25). Future screening of much larger libraries, quite feasible with the assays developed in this study, will help us to identify additional chemical scaffolds that can safely be optimized to target particular muscle types and syndromes. In parallel, we can pursue a modification of the compounds identified as modulating myosin ATPase. The compounds identified in this screen are already FDA-approved and in use as medications for several diseases, though some have significant side effects. These newly identified compounds affect muscle regulation at micromolar concentrations, in a range that should be accessible to optimization by medicinal chemistry to achieve greater specificity, higher activity, and reduce undesired side effects (Fig. 2).

## Experimental procedures

### Actin preparations and labeling

Actin was prepared from rabbit skeletal muscle by extracting acetone powder in cold water as described previously (13). For labeling, 50 μM of G-actin in 20 mM Tris pH 7.5, 0.2 mM CaCl_2_, 0.2 mM ATP was polymerized by the addition of 3 M KCl (to a final concentration of 100 mM) and 0.5 M MgCl_2_ (to a final concentration of 2 mM), followed by incubation at 23 °C for 1 h. Labeling with FMAL was done at a final FMAL concentration of 1 mM for 1 h at 23 °C. Labeling was stopped by the addition of a five-fold molar excess of DTT. Unincorporated dye was removed by cycling the actin through F-actin and G-actin states as described in (15). To avoid FRET between FMAL on the neighboring Cys-374 residues of actin monomers in F-actin, unlabeled G-actin was mixed with the FMAL-actin to achieve ∼10% FMAL-actin prior to the final actin polymerization. Finally, fluorescent-labeled F-actin was stabilized by the addition of equimolar phalloidin. Labeling of F-actin with Alexa Fluor 568 C_5_ maleimide (AF568, ThermoFisher Scientific) for the binding experiments with unlabeled cC0-C2 and fC1-C2 (Fig. S1) was done as described (13).

For all assays, F-actin was resuspended in and/or dialyzed against MOPS-actin binding buffer, M-ABB (100 mM KCl, 10 mM MOPS pH 6.8, 2 mM MgCl_2_, 0.2 mM CaCl_2_, and 0.2 mM ATP, 1 mM DTT).

### Recombinant human cMyBP-C fragment preparations and labeling

pET45b vectors encoding *E. coli* optimized codons for the cC0-C2 portion of human cMyBP-C with N-terminal 6x His tag and TEV protease cleavage site were obtained from GenScript. For FLT-FRET binding assays, we mutated cC0-C2 so that it contained a single cysteine at position 249, a surface-exposed residue in the C1 domain. To achieve this, four endogenous cysteines in cC0-C2 were mutated to amino acids found in other MyBP-C proteins leaving only one cysteine at position 249 (26). Protein production in *E. coli* BL21DE3-competent cells (New England Bio Labs) and purification of cC0-C2 protein using His60 Ni Superflow resin was done as described (27). cC0-C2 (with His-tag removed by TEV protease digestion) was further purified using size-exclusion chromatography to achieve >90% intact cC0-C2 as described (22) and then concentrated, dialyzed to 50/50 buffer (50 mM NaCl and 50 mM Tris, pH 7.5) and stored at 4 °C.

For FLT-FRET experiments, cC0-C2^Cys249^ was labeled with tetramethylrhodamine (TMR) in 50/50 buffer. cC0-C2^Cys249^ (50 μM) was first treated with the reducing agent TCEP (200 μM) for 30 min at 23 °C, and then TMR was added (from a 20 mM stock in DMF) to a final concentration of 100 μM. Labeling was done for 1 h at 23 °C and terminated by the addition DTT (to 1 mM). Unincorporated dye was removed by extensive dialysis against M-ABB buffer. To remove any precipitated dye and insoluble C0-C2, labeled C0-C2 was centrifuged 2× for 30 min at 100,000 RPM (350,000*g*) in a Beckman TLA-120.2 rotor. The degree of labeling was kept at 0.8 dye/cC0-C2 as measured by UV-vis absorbance.

The N-terminal domains from fMyBP-C, fC1-C2, were similarly expressed in bacteria and labeled with TMR. Labeling concentration of TMR for fC1-C2 was 150 μM.

### *In vitro* phosphorylation of cMyBP-C

cC0-C2 was treated with 7.5 ng PKA/μg cC0-C2 at 30 °C for 30 min, as described in (15, 22).

### Fluorescence data acquisition

Fluorescence lifetime (FLT) measurements were acquired using a high-throughput FLT plate reader (Fluorescence Innovations, Inc) (14, 27), provided by Photonic Pharma LLC. For FLT-FRET experiments, the donor FMAL-actin was excited with a 473-nm microchip laser (Bright Solutions) and emission was filtered with 488-nm long pass and 517/20-nm bandpass filters (Semrock). For FLT-TMR experiments, the same samples were excited at 532 nm. The observed waveforms were analyzed as described previously (15, 28).

Plates were also scanned using the spectral unmixing plate reader (SUPR; Fluorescence Innovations, Inc) to exclude fluorescent compounds, which can present as false positives in the FLT-FRET measurement (14, 29).

### FMAL-actin/TMR-MyBP-C FLT-FRET binding assays

FLT-FRET binding assays were performed as described (13) with the modification being that 0.25 μM F-actin labeled (to 10%) on Cys-374 with FMAL (donor) was incubated with cC0-C2^Cys249^, fC1-C2, or sC1-C2 labeled to 70 to 90% with TMR (acceptor) (for donor-acceptor data). The binding curve (Fig. 3) was generated from data from two separate preparations of actin cC0-C2^Cys249^ and fC1-C2. K_d_ values were determined by fitting the data to a quadratic model (Michaelis–Menten function) using Origin Pro 2019 software package through a nonlinear least-squares minimization (Levenberg–Marquardt algorithm). Control experiments detected no change in FLT of TMR C0-C2 due to its binding to unlabeled actin under the conditions of the primary screen, where actin is present at half the concentration of TMR C0-C2, so we do not expect this to affect the primary screen data.

### AF568-actin -MyBP-C FLT binding assays

AF568-Actin-MyBP-C FLT binding assays based on the change in the lifetime of AF568-labeled actin upon cC0-C2 or fC1-C2 (Fig. S1) were done as described (13).

### Selleck library screen

2684 Selleck compound (Cat# L1300-Z394273, mostly FDA-approved drugs, and active pharmaceutical ingredients) were received in 96-well plates and reformatted into 1536-well flat, black-bottom polypropylene plates (Greiner Bio-One). In total, 50 nl of each solution was dispensed in DMSO using an automated Echo 550 acoustic liquid dispenser (Labcyte). Compounds were formatted into the assay plates, at a final concentration of 30 μM, with the first two and second to last columns loaded with DMSO only (compound-free controls). In the final column, 30 μM of suramin was loaded as a positive control (tool compound). These assay plates were then heat-sealed using a PlateLoc Thermal Microplate Sealer (Agilent Technologies) and stored at −20 °C. Before screening, compound plates were equilibrated to room temperature (∼25 °C). 0.25 μM FMAL-actin without or with 0.5 μM TMR-cC0-C2 was dispensed into 1536-well assay microplates containing the compounds by a Multidrop Combi Reagent Dispenser (Thermo Fisher Scientific). Plates were incubated at room temperature for 60 min before recording the data with the fluorescence lifetime plate reader. Screens were performed in duplicates with independent preparations of actin and cC0-C2.

### FLT data analysis

Following data acquisition, time-resolved FLT waveforms observed for each well were fit by convolving the instrument response function (IRF) with a single-exponential decay to determine the FLT (τ) of the excited fluor (Equation 1) (15, 28, 30):

(1) $$\begin{array}{c} I\left(t\right)=F\left(t\right)\;*\;\text{IRF}\\ F\left(t\right)=I_{0}\;\exp\;\left(-t/\tau \right) \end{array}$$

where I is the measured waveform, *I_0_* is the fluorescence intensity upon peak excitation (*t* = 0) and τ is the FLT (t = τ when *I* decays to 1/e or ∼37% of *I*_*0*_).

FLT assay quality was determined by computing Z′ (Equation 2) as we have done previously (13). Z′ is indicative of the overall quality of the biosensor and a standard metric in screening to determine the suitability of an HTS assay (15, 16, 31):

(2) $$\begin{array}{l} \overset{\prime }{Z}=1-3\left(\frac{\sigma_{\text{FMAL}-\text{Actin}}+\sigma_{\text{FMAL}-\text{Actin}}{\;}_{\text{plus}\;\text{TMR}-\text{cC}0-C2}}{\left|\mu_{\text{FMAL}-\text{Actin}}-\mu_{\text{FMAL}-\text{Actin}}{\;}_{\text{plus}\;\text{TMR}-\text{cC}0-C2}\right|}\right) \end{array}$$

where σ_FMAL-Actin_ and σ_FMAL-Actin plus TMR-cC0-C2_ are the standard deviations, and μ _FMAL-Actin_ and μ _FMAL-Actin plus TMR-cC0-C2_ are the means of the FMAL-Actin lifetime in the absence and presence of the FRET acceptor TMR-cC0-C2, respectively. As a complementary measurement, Z′ was also computed with Equation 2 by comparing the statistical metrics for the FLT of FMAL-Actin plus TMR-cC0-C2 in the absence and presence of suramin (a compound blocking Actin-cC0-C2 interactions identified in our earlier study) (13). A Z′ of greater than 0.6 was achieved in this assay (see Table S1), with a value of 0.5 to 1 indicating an assay of excellent quality (15, 16, 31).

### HTS data analysis

After fitting the waveforms with a single exponential decay to quantify donor FLT, the change in FLT (Δτ) was computed by performing a moving median subtraction in the order the plate was scanned with a window size of half a plate row (24 columns). The reasons for this are twofold. Firstly, plate gradients are often observed due to the heating of the digitizer during acquisition. Second, performing Δτ computations with DMSO controls alone can sometimes be erroneous as the DMSO wells are on the edge of the plate, which occasionally exhibit artifacts due to processes needed for the preparation of the compound library being tested. As most compounds are likely to be non-hits, and therefore DMSO-like, computation of a moving median is an effective alternative to solving both gradient issues and edge-effect distortion of the primary metric for hit selection, Δτ. A complementary metric, FRET efficiency (E), was also computed as the fractional decrease of donor FLT (FMAL-actin, donor) in the absence and in the presence of acceptor (FMAL-actin+TMR-cC0-C2, donor-acceptor) as in Equation 3:

(3) $$E=1\;-\;\tau_{\text{donor}-\text{acceptor}}/\tau_{\text{donor}}$$

To account for the necessity to correct for plate gradients in the computation of E, the median τ_DMSO_ prior to the application of the moving median correction was added to each well’s Δτ value to recover a rescaled value of τ. As the screen was run in duplicate, the mean values of τ and Δτ were used in the computation of metrics for hit selection. Ηits that modulated the interaction of actin-cC0C2 were selected by computing the robust *z*-score (a measure of an individual compound’s effect on the biosensor with respect to the plate-wide or screen-wide statistics) on a plate-by-plate basis, with a hit threshold set at ±4. The robust z-score, where the median (*M*) and median absolute deviation (*MAD*) are used in place of the mean and SD (Equation 4), was used to best capture the most hits, as the standard z-score is more subject to strong outliers.

(4) $$z-\text{score}=\frac{\Delta \tau -\;M\left(\Delta \tau \right)}{MAD\left(\Delta \tau \right)}$$

Compounds that bound cC0-C2 and modified the fluorescence of TMR directly (measured by exciting TMR with a 532 nm laser source) were selected using identical criteria. Two potential sources of false positives are compounds that either affect the donor-only FLT directly or exhibit fluorescence under the conditions of the screen. Hits that exceeded the *z*-score hit criteria in the donor-only screen were excluded from consideration. To remove compounds with confounding fluorescence properties, a similarity index (SI, specifically the cosine distance, Equation 5) was computed using data from the SUPR instrument by comparing a region (500–540 nm) of the donor-only spectrum (*I*^(a)^) for each well to that of the plate-wide average DMSO spectrum (*I*^(b)^) in the same wavelength band (29). Compounds that exceeded an SI robust z-score of 4 were deemed likely fluorescent compounds and removed from consideration.

(5) $$SI=1-\;\frac{\sum I_{i}^{\left(a\right)}.\;I_{i}^{\left(b\right)}}{\sqrt{\sum {I_{i}^{\left(a\right)}\;.\;I}_{i}^{\left(a\right)}}\sqrt{\sum {I_{i}^{\left(b\right)}\;.\;I}_{i}^{\left(b\right)}}}$$

### Concentration–response assay

The primary hits were dissolved in DMSO to make a 10 mM stock solution, which was serially diluted in 96-well mother plates. Hits were screened at eight concentrations (0.5–100 μM). As indicated in the Figure 3 legend, FLT-FRET concentration-response assays were done with 0.25 μM FMAL-actin and 0.5 μM TMR-cC0-C2 and TMR-fC1-C2 and 1 μM PKA-phosphorylated TMR-cC0-C2. FLT-TMR concentration-response assays were done in the absence of actin with 0.5 μM TMR-labeled cC0-C2, PKA-phosphorylated cC0-C2, and fC1-C2. Compounds (1 μl) were transferred from the mother plates into 384-well plates using a Mosquito HV liquid handler (TTP Labtech Ltd, Hertfordshire, UK). The same procedure of dispensing as for the pilot screening was applied in the concentration-response assays. Concentration dependence of the FLT-FRET (E/E_DMSO_) or FLT-TMR (Δτ) change data was fit to a Hill equation only under certain conditions to help guide the eye (Fig. 4).

### Myofibril ATPase kinetics

Rabbit psoas and bovine cardiac myofibrils were prepared as described in our previous study (17). An enzyme-coupled, NADH-linked ATPase assay was used to measure skeletal and cardiac myofibril ATPase activity in 96-well microplates at 23 °C. Ionic strength was maintained below 0.2 M, so myofilaments remained insoluble and stable (32). To achieve optimal enzymatic activities, buffers for skeletal and cardiac myofibrils differed slightly (33, 34): skeletal in 50 mM MOPS, 100 mM KCl, 5 mM MgCl_2_, 1 mM EGTA, pH 7.0; cardiac in 20 mM MOPS, 35 mM NaCl, 5 mM MgCl_2_, 1 mM EGTA, pH 7.0. Each well in the microplate contained an assay mix of 0.84 mM phosphoenolpyruvate, 0.17 mM NADH, 10 U/vol pyruvate kinase, 20 U/vol lactate dehydrogenase. The concentration of free Ca^2+^ was controlled by EGTA buffering (35). Myofibrils were dispensed in microplates with a multichannel pipette and were incubated with assay mix plus compound of interest for 20 min at 23 °C. The concentration of myofibrils used was 0.01 mg/ml for skeletal and 0.05 mg/ml for cardiac myofibrils. The assay was started upon the addition of ATP, at a final concentration of 2.1 mM (total volume to 200 μl), and absorbance was recorded at 340 nm in a SpectraMax Plus microplate spectrophotometer from Molecular Devices. The steady-state myofibrillar ATPase rate was measured from NADH oxidation, measured as the rate of decrease in absorption at 340 nm for 30 min, with absorption data collected every 15 s. All data and statistical analysis of steady-state kinetics were conducted with the OriginPro program. The results were fitted with the Hill equation:

(6) $$V=V0+\left(\frac{Vmax}{1+1{0^{-n*}}^{\left(pCa50-pCa\right)}}\right)$$

where V is the ATPase rate and n is the Hill coefficient.

ATPase activity is reported on the μmol ATP/mg protein/min scale. Average data are presented as mean ± SD. Sample means are derived from three different myofibril preps (N = 3), each done in duplicate (n = 6).

The effects of compounds on ATPase activity of myofibrils were determined in the presence of 50 μM compound, across a 12-point pCa range from 4.0 (*activation*) to 8.0 (*relaxation*). The calcium sensitivity (pCa_50_) was determined by fitting the curves to the Hill equation. In all experiments, a 1% DMSO-only control was included. The normalized ATPase activity of the selected compounds can be found in Figure 6.

### Statistics

Average data are provided as mean ± standard deviation (SD) and each experiment was done with three separate protein preparations (N = 3) unless otherwise mentioned.

## Data availability

All data discussed are presented within the article.

## Supporting information

This article contains supporting information (13).

## Conflict of interest

D. D. T. holds equity in, and serves as President of, Photonic Pharma LLC. This relationship has been reviewed and managed by the University of Minnesota. The present research is a pre-commercial collaboration between Photonic Pharma, UMN, and the University of Arizona. B. A. C. serves as President of BC Biologics LLC. This relationship has been reviewed and managed by the University of Arizona. BC Biologics had no role in this study. B. A. C. filed a PCT patent application based on this work (patent pending, serial no. PCT/US21/14,142). The other authors declare no competing financial interests.
