# Supplementary material for: Drug discovery for heart failure targeting myosin-binding protein C
Source: J Biol Chem. 2023 Oct 20;299(12):105369. doi: 10.1016/j.jbc.2023.105369 (PMC10692721; doi:10.1016/j.jbc.2023.105369)
Supplement: Supporting Figures S1 and S2 and Tables S1 and S2 [file mmc1.docx]

Supporting information

**Drug discovery for heart failure targeting myosin-binding protein C**

**Thomas A. Bunch^1,^*, Piyali Guhathakurta^2,^*, Andrew R. Thompson^2^, Victoria C. Lepak^1^, Anna L. Carter^2^, Jennifer J. Thomas^3^, David D. Thomas^2,3,‡,^**^†^ **and Brett A. Colson^1,‡^** ^†^**.**

^1^Department of Cellular & Molecular Medicine, University of Arizona, Tucson, Arizona, USA

^2^Department of Biochemistry, Molecular Biology, and Biophysics, University of Minnesota, Minneapolis, Minnesota, USA

^3^Photonic Pharma LLC, Minneapolis, Minnesota, USA

*Equal contribution

‡Co-senior authors

†Corresponding authors

*Correspondence: David D. Thomas ([ddt@umn.edu](mailto:ddt@umn.edu))

Brett A. Colson ([bcolson@arizona.edu](mailto:bcolson@arizona.edu))

Running title: *High-throughput assay detecting cMyBP-C modulators*

**Keywords:** actin, cardiac muscle, cardiac myosin-binding protein C (cMyBP-C), contractile proteins, phosphorylation, protein kinase A (PKA), fluorescence lifetime (FLT), high-throughput screen (HTS), fluorescence resonance energy transfer (FRET), site-directed spectroscopy

| Table S1. FMAL-actin lifetime changes with TMR-cC0-C2 binding | | | | | | | |
| --- | --- | --- | --- | --- | --- | --- | --- |
| Screen |  | Average lifetime  (ns) | SD | CV (%) | n | Change FRET (%) | Z´ |
| 1 | NoTMR-cC0-C2 | 4.11 | 0.02 | 0.59 | 624 |  |  |
|  | WithTMR-cC0-C2 | 3.54 | 0.05 | 1.39 | 624 | 13.8 | 0.61 |
| 2 | No TMR-cC0-C2 | 3.90 | 0.02 | 0.57 | 624 |  |  |
|  | With TMR-cC0-C2 | 3.45 | 0.03 | 0.95 | 624 | 11.4 | 0.63 |
| Experiments were carried out with two separate protein preparations for FMAL-actin (0.25 µM, 10% labeled) without and with 2 separate TMR-C0-C2  protein preparations (0.5 µM, 70-90% labeled). The average FLT of FMAL, attached to F-actin (in ns), standard deviation (SD), coefficient of variance (CV), number of wells (n), change in FLT (FRET) due to TMR-cC0-C2, and Z´ factor are shown [N=2, n=1248]. | | | | | | | |

| **Table S2 Reproducible hits tested in Concentration Response Curves (CRC)** | | | | | | |
| --- | --- | --- | --- | --- | --- | --- |
| Compound |  | 473-nm Δτ (ns) | | 532-nm Δτ (ns) | | |
|  | Repeat 1 | Repeat 2 | z-score (based on avg Δτ) | Repeat 1 | Repeat 2 | z-score (based on avg Δτ) |
| Anidulafungin (LY303366) | 0.014 | 0.008 | 0.67 | 0.403 | 0.441 | 25.80 |
| Bedaquiline fumarate | -0.324 | -0.412 | -20.57 | -0.009 | -0.105 | -3.45 |
| Cefepime dihydrochloride monohydrate | 0.127 | 0.102 | 6.43 | 0.006 | -0.096 | -2.70 |
| Cefsulodin sodium | 0.394 | 0.316 | 19.91 | 0.114 | 0.083 | 6.07 |
| Chloroquine phosphate | -0.042 | -0.069 | -3.07 | 0.311 | 0.320 | 19.30 |
| Clindamycin palmitate HCl | -0.304 | -0.358 | -18.47 | -0.075 | -0.094 | -5.12 |
| Clinofibrate | 0.090 | 0.075 | 4.65 | 0.006 | 0.016 | 0.70 |
| Diammonium glycyrrhizinate | 0.077 | 0.080 | 4.42 | 0.022 | 0.017 | 1.22 |
| Elbasvir | 0.142 | 0.080 | 6.24 | -0.002 | 0.011 | 0.31 |
| Enoxaparin sodium | 0.190 | 0.190 | 10.68 | -0.023 | -0.033 | -1.67 |
| Erlotinib | 0.026 | 0.001 | 0.78 | 0.100 | 0.119 | 6.74 |
| Erlotinib HCl (OSI-744) | -0.040 | -0.010 | -1.36 | 0.138 | 0.020 | 4.88 |
| Ertapenem sodium | 0.353 | 0.231 | 16.37 | 0.079 | 0.157 | 7.23 |
| Erythromycin estolate | 0.149 | 0.101 | 7.01 | 0.002 | -0.031 | -0.86 |
| Febuxostat | 0.070 | 0.043 | 3.18 | 0.129 | 0.132 | 8.01 |
| Fenticonazole nitrate | -0.069 | -0.104 | -4.80 | -0.011 | -0.035 | -1.35 |
| Fidaxomicin | 0.057 | 0.084 | 3.98 | -0.001 | -0.028 | -0.86 |
| Fostamatinib (R788) | 0.110 | 0.046 | 4.40 | -0.027 | -0.063 | -2.72 |
| Hederagenin | 0.083 | 0.060 | 4.06 | -0.038 | -0.038 | -2.29 |
| α-Hederin | -0.087 | -0.051 | -3.83 | 0.105 | 0.175 | 8.58 |
| Heparin sodium | 0.440 | 0.363 | 22.49 | -0.052 | -0.051 | -3.09 |
| Laquinimod | 0.109 | 0.071 | 5.08 | -0.017 | -0.016 | -0.99 |
| Latamoxef sodium | 0.128 | 0.049 | 4.99 | -0.050 | -0.037 | -2.62 |
| Nilotinib hydrochloride | -0.063 | -0.031 | -2.59 | 0.121 | 0.105 | 6.94 |
| Paritaprevir (ABT-450) | 0.115 | 0.058 | 4.85 | 0.220 | 0.254 | 14.52 |
| Pneumocandin B0 | -0.050 | -0.053 | -2.83 | 0.179 | 0.149 | 10.04 |
| Pranlukast | 0.061 | 0.008 | 1.95 | 0.073 | 0.116 | 5.80 |
| Sacubitril/valsartan (LCZ696) | 0.140 | 0.095 | 6.60 | -0.025 | 0.012 | -0.37 |
| Saikosaponin D | -0.122 | -0.094 | -5.99 | 0.065 | 0.086 | 4.65 |
| Sanguinarine chloride | -0.105 | -0.086 | -5.31 | -0.160 | -0.020 | -5.46 |
| Scutellarin | 0.142 | 0.076 | 6.15 | -0.111 | -0.145 | -7.79 |
| Tamibarotene | 0.075 | 0.067 | 4.00 | 0.075 | 0.106 | 5.55 |
| Tocofersolan | -0.166 | -0.159 | -9.06 | 0.072 | 0.065 | 4.23 |
| Triclocarban | -0.128 | -0.042 | -4.72 | 0.374 | 0.415 | 24.12 |
| Troxerutin | -0.024 | -0.024 | -1.32 | 0.074 | 0.088 | 4.99 |
| Valsartan | 0.149 | 0.058 | 5.83 | 0.000 | 0.013 | 0.44 |
| Verteporfin | 0.093 | 0.064 | 4.41 | 0.065 | 0.075 | 4.32 |
| Zaltoprofen | 0.113 | 0.083 | 5.52 | -0.015 | 0.015 | 0.04 |
| **Doxazosin** | -0.266 | -0.231 | -13.87 | 0.125 | 0.126 | 7.70 |
| **Eltrombopag olamine** | 0.270 | 0.162 | 12.13 | -0.267 | -0.418 | -20.87 |
| **Flopropione** | -0.335 | -0.210 | -15.21 | -0.062 | -0.073 | -4.10 |
| **Lumiracoxib** | 0.093 | 0.064 | 4.45 | -0.054 | -0.036 | -2.72 |
| **Luteolin** | -0.309 | -0.214 | -14.58 | -0.277 | -0.302 | -17.63 |
| **Masitinib (AB1010)** | -0.336 | -0.197 | -14.87 | -0.071 | -0.082 | -4.66 |
| **Olsalazine Sodium** | 0.202 | 0.096 | 8.38 | -0.057 | -0.123 | -5.46 |
| **Oxytetracycline (Terramycin)** | 0.175 | 0.122 | 8.34 | -0.019 | -0.057 | -2.29 |
| **Proanthocyanidins** | -0.370 | -0.436 | -22.49 | -1.202 | -1.301 | -76.37 |
| **Regorafenib (BAY 73-4506)** | -0.029 | -0.026 | -1.50 | 0.099 | 0.102 | 6.17 |
| **Telotristat etiprate (LX 1606 Hippurate)** | -0.136 | -0.122 | -7.19 | 0.064 | 0.093 | 4.83 |
| **Proflavine hemisulfate** | 0.430 | 0.337 | 21.48 | -0.016 | -0.080 | -2.91 |
| **Protoporphyrin IX** | 0.144 | 0.076 | 6.21 | 0.067 | 0.016 | 2.58 |
| **Pyrantel pamoate** | 0.313 | 0.250 | 15.79 | -0.043 | -0.100 | -4.32 |
| **Raloxifene** | -0.490 | -0.307 | -22.27 | -0.025 | -0.147 | -5.22 |
| Regorafenib HCl* |  |  |  |  |  |  |
| Ampicillin sodium | 0.184 | 0.161 | 9.70 | 0.003 | 0.007 | 0.34 |
| Benzonatate | 0.108 | -0.001 | 3.05 | -0.101 | -0.124 | -6.82 |
| Hyodeoxycholic acid (HDCA) | 0.111 | 0.072 | 5.17 | -0.021 | -0.120 | -4.27 |
| Relugolix | 0.366 | -0.004 | 10.15 | -0.169 | -0.015 | -5.58 |
| Thonzylamine | 0.012 | -0.449 | -12.17 | -0.034 | -0.382 | -12.66 |
| 8-Hydroxyquinoline | -0.025 | -0.188 | -5.91 | -0.167 | -0.285 | -13.76 |
| The 60 hits identified in the initial screen. Compounds were selected by first computing the mean Δτ response between two replicates, followed by computing the robust z-score using the plate-wide statistics (computed on the mean Δτ response between replicates). The hit threshold was set at a robust z-score of ±4. Compounds in **bold** affected the fluorescence lifetime (FLT) of FMAL-actin at higher concentrations and were therefore excluded from functional assays. The last six compounds did not show any FRET change upon retesting. *Not tested due to lack of availability. | | | | | | |

**Fig. S1. Suramin, NF023 and Aurintricarboxylic acid (ATA) inhibit fC1-C2 binding to actin**. Suramin (20 µM), NF023 (50 µM) and ATA (20 µM) decreased the binding of fC1-C2 (2 µM, red) to Alexa Fluor 568-actin (1 µM). The effects on cC0-C2 (black) as described in (13) are shown for comparison. Values are the % decrease in FLT of Alexa Fluor 568-actin due to cC0-C2 or fC1-C2 as described previously (13), and are the average ± SD. n=3-5.

**Fig. S2. Compound effects on TMR-MyBP-C lifetime.** Suramin, NF023 and Aurintricarboxylic acid (ATA) change the FLT of TMR on cardiac C0-C2 and skeletal C1-C2. cC0-C2, sC1-C2S (slow skeletal short form), sC1-C2L (slow skeletal long form), and fC1-C2 were labeled with TMR whose FLT in the absence of compound was 2.30-2.64 ns (black). Long and short skeletal forms are the same sC1-C2 protein fragment except that the long form contains an insertion of 25 amino acids to represent an alternative splicing of slow skeletal MyBP-C as present in humans. Suramin (20 µM, red) and NF023 (50 µM, green) increased the FLT of TMR-MyBP-C, while ATA (20 µM, blue) decreased FLT for all MyBP-C isoforms. **A.** FLT of TMR-MyBP-C (in ns) without and with compounds is shown. **B.** The percent change in FLT compared to DMSO control (no compound) is displayed. All values are the average ± SD. n=5.
